# Towards Ending AIDS: The Additional Role of HIV Self-Testing in Thailand

**DOI:** 10.9745/GHSP-D-24-00156

**Published:** 2024-12-20

**Authors:** Cheewanan Lertpiriyasuwat, Patsaya Mookleemas, Naparat Pattarapayoon, Darinda Rosa, Viroj Tangcharoensathien

**Affiliations:** a Division of AIDS and STIs, Department of Disease Control, Ministry of Public Health, Nonthaburi, Thailand.; b International Health Policy Program, Ministry of Public Health, Nonthaburi, Thailand.

## Abstract

**Background:**

In 2022, 10% of an estimated 560,000 people living with HIV in Thailand were unaware of their HIV status. A well-established HIV program is a solid platform for integrating HIV self-testing (HST) as part of efforts to end AIDS. We analyzed how HST was integrated into the national HIV program and became a benefit package.

**Policy Adoption of HST:**

In 2015, the National AIDS Prevention and Alleviation Committee included HST as a strategy to end HIV/AIDS by 2030. This led to collaboration between the Department of Disease Control (DDC), Food and Drug Administration (FDA), and partner networks, including civil society organizations, to amend policy regulations, allowing HIV testing outside health care facilities and facilitating HST registration. By 2024, 4 HST commercial products were registered by the Thai FDA.

**Program Pilots:**

In 2020, the DDC launched pilot programs distributing HST kits through private pharmacies in Bangkok and online platforms. Preliminary findings showed feasibility in reaching key populations and adolescents. In 2023, HST was included in the Universal Health Coverage benefit package, providing free access to all citizens. Guidelines, e-learning, public awareness campaigns, and a reimbursement system of HST were developed and implemented. By September 2024, over 166,000 users had received HST kits.

**Lessons Learned:**

Leadership, scientific evidence, feasibility testing through pilots, regulatory adjustments, licensing, price negotiations by the National Health Security Office, and stakeholder and community engagement were key to the program’s success. A nationwide distribution network through public and private health care facilities, including pharmacies, was a key enabling factor for HST delivery.

**Conclusions:**

HST is an additional intervention to increase awareness of HIV status and a key component in Thailand’s effort to end HIV/AIDS.

## Abstract (precis)

HIV self-testing is an additional intervention to increase individuals’ awareness of their HIV status and a key component in Thailand’s effort to end HIV/AIDS.

## Key Messages

- In 2015, Thailand introduced HIV self-testing (HST) into its national HIV program, following World Health Organization recommendations for safe, accurate, and accessible testing for individuals who may avoid health care facilities for fear of stigma. HST aims to increase awareness of HIV status and prompt treatment across the population.
- In 2023, the National Health Security Board included HST in the universal health coverage package, offering it free of charge with no annual limits on the number of tests.
- Program managers provided comprehensive information on HST, including how to interpret results and next steps.
- The National Health Security Office negotiated for the lowest price with assured quality and the national HIV program established a monitoring system and conducted impact assessment to enhance program performance.

## BACKGROUND

In 2022, 10% of an estimated 560,000 people living with HIV (PLHIV) in Thailand were unaware of their HIV status,^1^ a slight improvement from 11% in 2015. However, the percentage of key populations, such as men who have sex with men (MSM), sex workers, transgender and gender diverse people, people who inject drugs, prisoners, and other incarcerated people,^2^ who were unaware of their HIV status remains unknown. In 2018, more than half of new HIV infections annually were among key populations, particularly MSM and transgender women.^3^ In 2022, 55% of new infections in Thailand occurred among key populations and their partners, compared to 44% in 2010.^4^ Providing access to HIV testing to raise awareness of HIV status and immediate enrollment in antiretroviral therapy (ART) is crucial for ending HIV/AIDS, highlighting the need for policies that improve access to testing while minimizing stigma.

HIV self-testing (HST) can play a role in increasing the awareness of HIV status among the general population and key populations, facilitating either continued prevention or enrollment in an ART program for those confirmed positive. In 2016, the World Health Organization recommended HST as a safe, accurate, and effective way to reach people who might not otherwise get tested. It was found that lay users could reliably and accurately perform HST, achieving test results comparable to those of trained health care workers.^5^ Nearly 100 countries now incorporate HST into their national testing strategies.^6^

A global systematic review and meta-analysis supported the role of HST, showing that the linkage to care was similar between the HST arm and standard care arm with an effect size of 0.92 (95% confidence interval=0.45, 1.86).^7^

Policymakers must ensure that ART services are accessible and free from stigma and discrimination. Thailand’s universal ART program is well-developed. By 2022, 90% of the estimated 507,009 PLHIV who knew their status were on ART, and 97% of them had achieved viral suppression.^1^ Universal ART is provided free of charge to all PLHIV. Also, post-exposure and pre-exposure prophylaxis have been integrated into the Universal Health Coverage (UHC) policy since 2022 and 2020, respectively. This article reviewed international and domestic HST experiences and analyzed how HST was introduced and integrated into Thailand’s HIV program, focusing on regulation amendment and health system preparation for HST implementation.

This article analyzed how HST was introduced and integrated into Thailand’s HIV program, focusing on regulation amendment and health system preparation for HST implementation.

Implementation science studies the methods to promote the uptake of research findings into practice.^8^ Although the field has grown over the past decade, its application to policy remains limited.^9^ This study bridges implementation science and policy decision-making, using HST as a policy tracer to explore how evidence from HST pilots in Thailand and abroad informed policymakers in scaling up HST at the national level.

## UNDERSTANDING INTERNATIONAL AND DOMESTIC EXPERIENCES WITH HIV SELF-TESTING IMPLEMENTATION

To understand the global and national situation, HST acceptability, effectiveness, and implementation experiences, first, 3 authors (CL, VT, and NP) reviewed international and national literature on HST. Second, CL, NP, PM, and DR reviewed 16 documents related to policy adoption, system preparation, and operational aspects of HST, including meeting reports, Food and Drug Administration (FDA) regulations, HST product profiles, costs, and performance reports. CL, VT, NP, and PM synthesized the findings to understand how evidence from international experiences and domestic implementation science influenced the adoption of HST. Additionally, we held 3 brainstorming sessions in 2024 with national HIV program managers and key personnel involved in HST program development and implementation to identify lessons learned and provide recommendations. The costs of HST products that we present in this article were converted from Thai Baht to US$ using an exchange rate of 34.6 Baht per US$ as of May 28, 2024.

### International Experiences

The high cost of the oral HST kit, OraQuick, with a retail price ranging from US$40–46, remains a major barrier to wider adoption in low- and middle-income countries. Additional barriers include overly complex instructional materials for some populations and distribution programs of unknown efficacy.^10^

We reviewed international experiences with different HST rollout and uptake approaches in both general and key populations.^6^^,^^7^^,^^11^^–^^15^ We found that these experiences were context specific, especially the extent to which their health systems could provide support, such as follow-on confirmation tests and access to ART services, as well as social and cultural norms in accepting ART, economic and affordability by insurance or households, and regulatory ecosystems for licensing HST. International experiences cannot be directly applied to countries without context-specific adjustments.

Innovative diagnostic technologies, such as point-of-care tests like HST, blood sugar tests, and the recent COVID-19 antigen test kits, can ease pressure on overburdened health care laboratory services, provided that HST is linked to confirmation testing and care.^16^

### Thailand’s Policy Adoption of HIV Self-Testing

Before deciding to introduce HST in Thailand, several issues had to be addressed, such as perceptions and willingness to use HST among general and key populations, availability of follow-on counseling and supervision, ability to perform self-testing accurately, product sensitivity and specificity, cost and affordability by insurance or households, and the concordance between HST and confirmed tests by health care workers.^17^^,^^18^

Although the U.S. Food and Drug Administration registered the first over-the-counter HST kit in 1996, HST was not adopted in Thailand due to legal constraints on HIV testing. In 2009, Notification of the Ministry of Public Health (MOPH),^19^ in accordance with Article 6 of the 2008 Medical Device Act (2551BE), mandated that health facility-based HIV screening tests could be sold and tested at health care facilities with 100% sensitivity and 99.5% specificity. At that time, no HST products were available in Thailand. Recognizing HST’s potential to improve awareness and testing among key populations and that 10% of all PLHIV were unaware of their HIV status, the Department of Disease Control (DDC) initiated policy measures to advocate for HST adoption.

In July 2013, a consultative meeting with HIV experts who were doctors and laboratory technicians, representatives of the PLHIV network, and civil society organizations (CSOs) was held to discuss the use of an oral fluid-based rapid HIV test kit, given its sensitivity and specificity. Following the meeting, the DDC submitted a formal letter to the Thai Food and Drug Administration (FDA) in August 2013, advocating for amending the Notification of the MOPH related to HIV testing to include a self-testing option.

In May 2014, a national subcommittee on medical biotechnology in HIV prevention was established, bringing together HIV experts, representatives of the PLHIV network, CSOs, the Thai FDA, the Division of AIDS and STIs (DAS) under the DDC, and the National Health Security Office (NHSO) to trigger the legal amendment process. In 2015, the Thailand National AIDS Prevention and Alleviation Committee (NAC) increased access to HST as one of the strategies for ending HIV/AIDS, as committed to in the 2030 Sustainable Development Goals, and designated the MOPH to assess the feasibility of HST rollout. The DAS, in collaboration with other stakeholders, such as the Thai FDA and the Department of Medical Sciences (DMSC), explored the application of HST for HIV screening. This initiative aligned with the World Health Organization’s 2016 HST guidelines.^20^

The Thai FDA’s internal process of amending the Notification of MOPH involved collaboration between the Thai FDA, the DMSC, and a multidisciplinary group of HIV experts in the field of HIV testing, prevention, and treatment, including doctors, nurses, medical technologists, and pharmacists. These experts were responsible for developing standards for the assessment of HST in Thailand. Amendment of regulations in accordance with the Medical Device Act took several years, as the process involved many actors and bureaucratic inertia. Finally, in 2019, a Notification of MOPH was published,^21^ allowing HST kits to be registered for sale and used by individuals outside health care facilities to promote early detection and access to treatment and support efforts to end AIDS.

The chronological events that informed the policy decision to amend the regulation, integrate HST into the UHC benefit package, and initiate pilot programs are described in the Figure.

**FIGURE:**
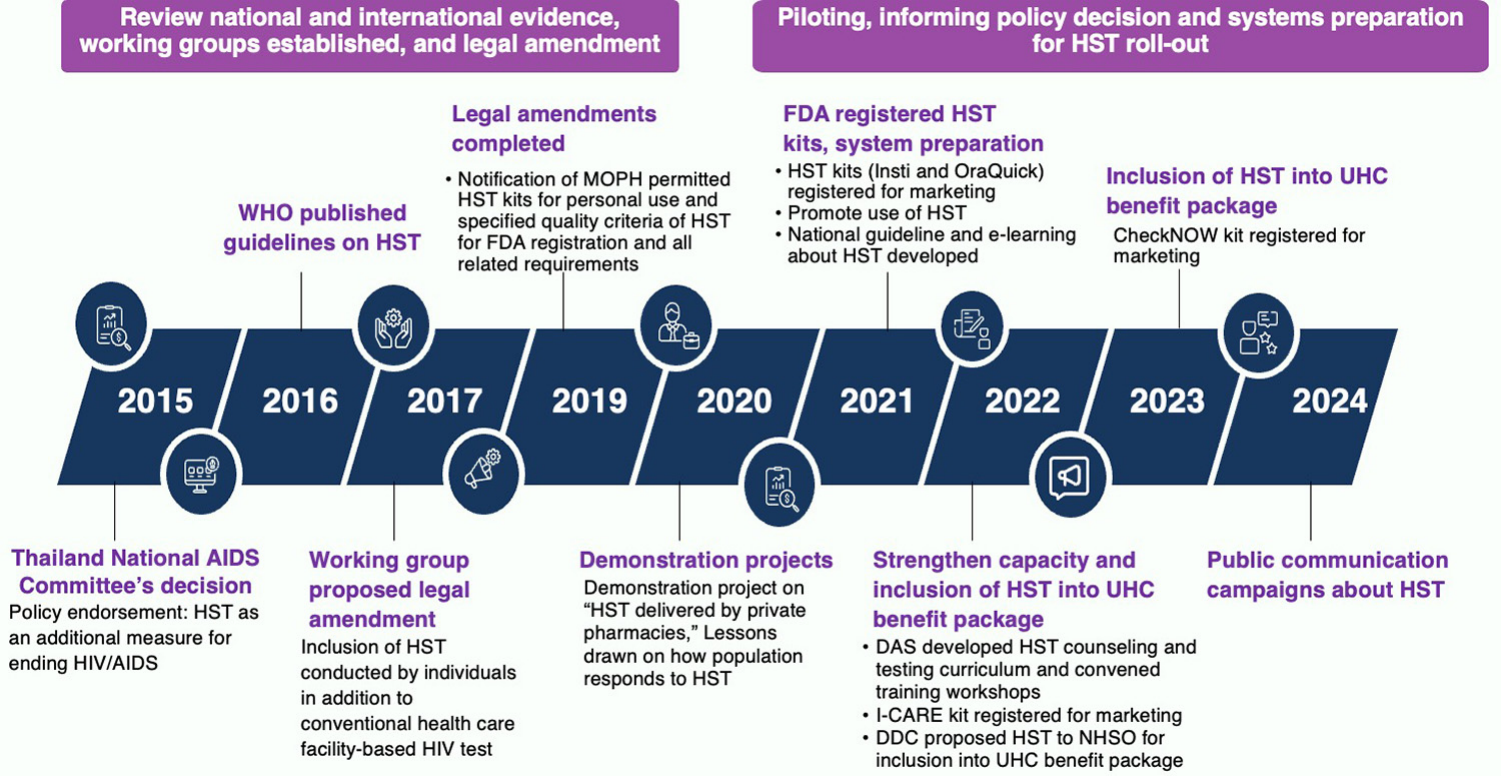
Events That Informed the Policy Decisions to Integrate HIV Self-Test Into the Universal Health Coverage Benefit Package in Thailand Abbreviations: DAS, Division of AIDS and STIs; FDA, Food and Drug Administration; HST, HIV self-test; MOPH, Ministry of Public Health; NHSO, National Health Security Office; UHC, universal health coverage; WHO, World Health Organization.

The HST adoption was influenced by 2 drivers: (1) scientific evidence, including international literature and evidence from implementation science through various pilots in different population groups; and (2) the government’s commitment towards ending AIDS, recognizing HST as a potential measure to increase awareness of HIV infection. Extensive geographical coverage of public and private health care delivery systems was a key enabler for rapid rollout.

The HST adoption was influenced by evidence from various pilot experiences and the government’s commitment toward ending AIDS, recognizing HST as a potential measure to increase awareness of HIV infection.

In Thailand, evidence of cost-effectiveness is required for new interventions or medicines to be included in the UHC benefit package or the National List of Essential Medicines. This ensures efficient resource use. In addition to cost-effectiveness, considerations include equity, feasibility, budget impact, and financial sustainability.^22^ Laboratory-based HIV testing was integrated into Thailand’s HIV program in the 1990s as a key measure in the national response, during which the HIV incidence reached its peak in 1992, with approximately 115,000 cases reported during the most severe period of the epidemic.^23^ Laboratory-based HIV testing was de facto adopted without considerations of cost-effectiveness evidence. Similarly, no cost-effectiveness study was conducted and used for HST adoption.

## HIV SELF-TESTING PRODUCT LICENSING AND REQUIREMENT

This policy shift, which allowed HST to be done by individuals, ended the previous regulation that limited HIV testing to health care facilities. Because HST is a self-test, no prescription is required, and it can be sold to any individual. Several HSTs are already available on the market. The 2019 Notification of MOPH outlined the required sensitivity and specificity levels for FDA approval of HST (Table 1); outlined the quality and standards for registration, including packaging, usage instructions, storage, warnings, and the test’s detectable range; and required an insertion of a user’s self-assessment form for HIV risk and QR codes linking to counseling and ART services.

**TABLE 1.** Testing Criteria for HIV Tests According to Notification of MOPH

**Testing criteria**	**Laboratory-Based HIV Diagnostic Tests (Professional Use)**		**HIV Self-Testing Kits**	
MOPH notification	2009		2019	
	Diagnostic Sensitivity	Diagnostic Specificity	Diagnostic Sensitivity	Diagnostic Specificity
Blood or blood component testing	Not less than 99.50%	Not less than 99.00%	Not less than 99.50%	Not less than 99.00%
Oral fluid testing			Not less than 99.00%	Not less than 98.00%

Abbreviation: MOPH, Ministry of Public Health.

In 2021, the Thai FDA registered the first 2 HST brands, Insti (using fingertip blood) and OraQuick (using oral fluid), which were sold in private pharmacies in select provinces. Subsequently, I-CARE (fingertip blood) and CheckNOW (fingertip blood) were registered in 2022 and 2023, respectively. Although there are 4 brands available, their prices range from 90 to 700 Baht (US$2.6–20.3) per kit, which remains unaffordable for many people (Table 2).

**TABLE 2.** Technical Profiles of Four HST Commercial Products Registered in the Thai Market by 2024^a^

**Product Profiles**	**Insti**	**OraQuick**	**I-CARE**	**CheckNOW**
Specimen type	Fingertip blood	Oral fluid	Fingertip blood	Fingertip blood
Principle of test kit	Immuno Dot-Flow-through device	Immuno-chromatography (2^nd^ generation)	Lateral flow immunoassay	Immuno-chromatography (3^rd^ generation)
Sensitivity as declared on the documentation (sensitivity required by MOPH), %	99.50 (99.50)	100 (99.00)^b^	99.50 (99.50)	100 (99.50)
Specificity as declared on the documentation (specificity required by MOPH), %	100 (99.00)	99.87 (98.00)^b^	100 (99.00)	99.90 (99.00)
FDA registration date	May 28, 2021	July 20, 2021	September 13, 2022	October 25, 2023
Retail market price, Baht (US$)^c^	590–700 (17.10–20.30)	350 (10.10)	300 (8.70)	90 (2.60)
E-bidding price by DDC, Baht (US$)	200 (5.80)	150 (4.3)	135 (3.9)	80 (2.3)
Distribution in the market	334 pharmacies in 39 provinces and online channels	252 pharmacies in 56 provinces and online channels	Online channels only	Service units registered with NHSO

Abbreviations: DDC, Department of Disease Control; FDA, Food and Drug Administration; HST, HIV self-testing; MOPH, Ministry of Public Health; NHSO, National Health Security Office.

^a^ Data as of May 28, 2024.

^b^ Thailand Food and Drug Administration sets a slightly lower sensitivity and specificity for oral fluid test than blood test.

^c^ Exchange rate as of May 28, 2024.

In the initial phase of rollout, these certified kits were made available through the DDC under the MOPH. Consequently, there was a concerted effort of stakeholders from the DDC, the DMSC, the NHSO, the Thai FDA, HIV experts, PLHIV network, CSOs representing key populations, and international organizations, such as the Joint United Nations Programme on HIV/AIDS to advocate for the inclusion of HST in the UHC health benefit package. This initiative aimed to enhance accessibility to free HIV testing among the Thai population, particularly among key populations who may have faced barriers to traditional testing or preferred the convenience of self-testing. Enabling private, at-home HIV testing aimed to increase awareness of HIV status and facilitate timely access to care and support services.

## HIV SELF-TESTING PROGRAM PILOTING

To support policy development, several studies^24^^–^^28^ assessed the feasibility of introducing and integrating HST into Thailand’s HIV program, exploring delivery models and affordable pricing.

In 2022, the DAS conducted a feasibility study of HST delivery in the Bangkok Metropolitan area, focusing on MSM and transgender individuals, and supported by a Thailand MOPH–U.S. Centers for Disease Control and Prevention Collaboration (TUC). A digital platform, “Buddy Station,” was launched, allowing users to register for OraQuick test kits that were available for pick-up at 36 private pharmacies in Bangkok or via postal delivery. The platform also provided result reporting, counseling, confirmatory testing, and links to ART services. Preliminary results showed that of 3,537 participants, 1,490 (42.1%) picked up a HST kit, and 618 (41.5%) reported their results: 14 were positive, 519 negative, and 4 invalid. Notably, 49.0% were first-time testers, with participants emphasizing convenience (89.8%), privacy (81.4%), confidentiality (72.0%), and ease of use (70.9%). The study confirmed the feasibility of HST delivery through private pharmacies.

In 2022, the DAS and Siriraj Hospital launched the “Stand by You” project, promoting the use of HST and providing free HST kits to MSM adolescents in educational institutions and other organizations in the Bangkok Metropolitan area via a website or a line official account, with support from the TUC, Thai government, and a private company. By September 2024, the number of users accessing HST kits increased, not only among MSM adolescents but also among other populations nationwide. Data showed that 9,272 kits were delivered by post, and the results of 8,135 users (87.7%) were reported. Of these, 7,433 (91.4%) were negative, and 287 (3.5%) were positive. All positive cases were referred for confirmatory testing and treatment at ART clinics. This pilot demonstrated the acceptability of HST among adolescents and the suitability of online platforms for reaching them.

To study various models of HST service delivery, the DDC piloted HST in 2023 in 4 provinces: Chiang Mai, Surat Thani, Chonburi, and Nakhon Ratchasima. By September 2024, 9,560 participants picked up test kits, but only 49.9% reported their results: 43 positive cases (0.9%), 4,672 negative cases (98.0%), and 54 invalid cases (1.1%).

These pilots tested the feasibility of different pickup locations, such as from subdistrict hospitals, provincial health offices, clinics, universities, or private pharmacies, or ordering from online platforms and delivery by mail. The findings informed nationwide program designs to effectively reach as many populations as possible. The pilots also captured HIV-positive individuals and referred them for confirmatory testing, treatment, and care. However, the positive rates were low, raising concerns about underreporting and difficulties in reaching hard-to-reach populations, who may have higher rates of positive results. To address these issues, efforts must focus on ensuring information access, linking users to confirmatory tests and ART, and engaging CSOs and peers to enhance outreach.

Challenges persisted, particularly the stigma and discrimination experienced by key populations. Advocacy is needed to normalize HIV testing and reduce barriers, along with extensive public education and dissemination of knowledge about HST to foster greater awareness and understanding among the general population. The success of these initiatives relied on collaboration among the government, private sector, and CSOs, contributing to the research outcomes.

## PREPARATION FOR SYSTEMS READINESS FOR HIV SELF-TESTING IMPLEMENTATION

In 2020, the NAC requested the DDC prepare a system to support HST implementation and promote public awareness of HIV self-screening. In 2021, the DAS, being cognizant of the importance of strengthening knowledge and techniques for using HST among the general population, developed and launched a national guideline on HIV self-screening test service^29^ and e-learning for the use of HST.^30^

In 2022, the DAS developed a curriculum for counseling and testing, offering training through online and onsite courses for professional nurses, counselors, and CSOs. This training aimed to ensure that counselors could effectively assist individuals using the self-tests. A total of 475 participants were trained during 2022–2024, with invitations extended through Provincial Public Health Offices, Regional Offices of Disease Prevention and Control, health care facilities, and CSOs.

On June 15, 2022, the NAC approved a proposal submitted by the DDC and partners to scale up HST nationwide, fully subsidized by the government through the NHSO. In October 2022, the DDC appointed a working group representing various government agencies, the NHSO, private sector, and civil society, to enhance access to HST. This move aimed to enhance the coordination and nationwide implementation of HST. After 2 meetings of the working group, several recommendations were made about integrating HST into the national health insurance benefit package related to HIV prevention, establishing delivery models, ensuring links to confirmatory testing and treatment, and strategies to increase voluntary, confidential access to HST.

In November 2022, the DDC took a further step by submitting a letter to the NHSO Secretary General proposing the inclusion of HST in the HIV prevention benefit package, emphasizing the need for wide access, affordability, and long-term financial sustainability. In March 2023, the National Health Security Board assessed the impact and advantages of implementing the HST, noting that it improved self-identification of HIV status and reduced workload and congestion in hospital-based testing. Because HST could replace facility-based testing without requiring additional budget, the Board approved its inclusion in the prevention benefit package, fully subsidized by the government, effective April 1, 2023. The Board also mandated the NHSO to negotiate prices and ensure rapid rollout. A comprehensive service guideline for HST was approved, including data systems and reimbursement mechanisms. The program design aimed to enhance universal and convenient access to HST nationwide.

In March 2023, the National Health Security Board assessed the impact and advantages of implementing the HST, noting that it improved self-identification of HIV status and reduced workload and congestion in hospital-based testing.

As of August 2023, the NHSO published service reimbursement details. All Thai citizens, including key populations of all ages, can use an unlimited number of fully subsidized HST kits annually. Public hospitals, designated private health care facilities, and primary health care units that are registered with the NHSO serve as delivery sites. These sites purchase HST kits, distribute them to users, and submit claims for reimbursement of 100 Baht (US$2.9) per kit from the NHSO. The reimbursement amount is based on the lowest market price, with CheckNOW offering 80 Baht (US$2.3) per test through electronic bidding (Table 2). The reimbursement rate is lower than the market prices of 3 of 4 registered HST products. The reimbursement rate is affordable for the NHSO, and the 100 Baht reimbursement provides a small margin that incentivizes health care providers registered with the NHSO to offer HST to the population.

As of October 2023, only 67 health facilities had procured and provided HST kits to 6,500 users and received reimbursement from the NHSO. The DAS also provided free HST kits to participants in pilot implementation programs and research studies. Among 6,500 users, 40 users received more than 1 kit. Data from October 2023 to September 18, 2024, indicated that since the availability of a fourth product priced at 90 Baht (US$2.6), approximately 186,408 HST kits have been distributed to 166,851 users.

To address concerns about the stigma associated with receiving HST from health care facilities, the NHSO expanded HST provision to CSOs, laboratory clinics, and private pharmacies starting on October 1, 2024, maintaining the same reimbursement rate of 100 Baht per test. This extension aims to better reach key populations, as CSOs have strong networks with communities.

To promote public awareness of HST, the DAS delivered 32,100 and 33,700 HST kits to prisoners at entry and exit in 2021 and 2022, respectively, with support from the Global Fund. Additionally, in 2022, HST kits were provided to all sexual partners of PLHIV who visited health facilities, enrolling them in the HIV Dual Screening Program, which uses HST for partners of PLHIV. There are no available data on positivity rates, as users conduct the test privately and are not required to report their results.

Public communication campaigns and HST promotion through online media channels, such as Facebook, TikTok, and Twitter, also closely engaged key populations, including MSM and adolescents, to raise awareness and encourage testing. Campaigns focused on empowering individuals to interpret HST results and understand the next steps based on positive or negative results. A few websites were developed by partners and the DAS for HST delivery and confidential counseling. In 2024, the DAS launched online counseling services for users seeking support before and after using HST kits.^31^

The DAS promotes these websites within key population networks and CSOs. Video clips demonstrating proper HST techniques were also released. HST serves as a tool for normalizing HIV testing, offering advantages such as ease of use, reduced stigma, universal access, and affordability through the NHSO, ultimately leading to increased testing and ART enrollment for those who test positive while helping others maintain their negative status.

## DOES HIV SELF-TEST WORK?

Initially, we found that HST is effective. The product profiles by the FDA for licensing require high sensitivity and specificity, ensuring accuracy. Various pilot studies indicate that users can manage, use, and interpret HST results properly. Between October 2023 and September 18, 2024, a total of 186,408 HST kits were used, with the NHSO subsidizing each test at 100 Baht (US$2.9), making it affordable and financially sustainable. However, additional evidence is needed regarding the proportion of HST-positive individuals enrolled in the universal ART program.

## IMPLEMENTATION CHALLENGES

We identified several challenges in implementing HST. Many health care providers hesitated to order and stock HST kits due to unpredictable demands, which could lead to expiration and financial loss. Price and volume were interlinked; higher prices were often imposed for smaller purchases, and logistical costs rose in remote areas with limited demand. The price of HST kits in the Thai market remained high primarily due to the oligopoly of 4 licensed suppliers who had more power to control prices than purchasers.

Although public awareness of HST has increased, it is not widespread. Some individuals continued to purchase HST kits online from unlicensed suppliers, raising concerns about test accuracy, and disliked the testing methods, such as fingerstick sample collection, or lacked sufficient information. These incidents have contributed to the spread of misinformation on social media, negatively impacting public confidence in HST.

To overcome these challenges, it is essential to enhance public awareness and demand for HST while monitoring and debunking misinformation. The NHSO can leverage its monopsonistic purchasing power as a single buyer representing all health care providers in the country. This enables the NHSO to negotiate the lowest possible prices while ensuring quality, allowing each health care provider to purchase HST products at the negotiated price.

## LESSONS LEARNED

Meaningful participation by stakeholders at all stages of the program rollout ensured ownership and commitment. Stakeholders included the NAC, relevant government agencies, academia, and CSOs (e.g., the Institute for Research and Innovation in HIV, FHI360, the Rainbow Sky Association, the Foundation for AIDS Access, and the HIV/AIDS Network of Thailand), as well as representatives from key populations (e.g., sex workers and MSM), the NHSO, and health care providers (including private pharmacies). These stakeholders were actively involved in program initiation, public awareness campaigns, pilot testing, training through online platforms and social media, delivery of HST, counseling, service navigation, system design, and implementation. Meaningful participation was also evident in the involvement of numerous key population-led organizations and key population-led services. Their engagement formed a strong foundation for HST policy development and implementation in Thailand.

Meaningful participation by stakeholders at all stages of the program rollout ensured ownership and commitment.

The following key lessons were learned by integrating HST into the HIV/AIDS program in Thailand.

- Early engagement with all stakeholders: Insights from health care providers, policymakers, and CSOs from the start can help identify regulatory hurdles and facilitate the approval process.
- Proactive addressing of misconceptions: Targeted educational campaigns can dispel myths about self-testing, such as fears of suicide following a positive result. Providing clear information about available counseling and support after HST can help build public trust and acceptance.
- Streamlining of regulatory processes: Advocating for regulatory amendments to allow HST options alongside standard health facility laboratory settings should include highlighting successful models from other countries to demonstrate feasibility.
- Access to follow-up care: Ensuring services for confirmatory testing and ART are linked to self-testing is critical. Partnerships with local health care facilities can facilitate seamless access to these services. Thailand’s universal ART program already covers 470,000 (82%) of the estimated 580,000 PLHIV.^32^
- Leveraging technology: Digital platforms can provide essential information and support, making it easier for individuals to navigate the testing process and access resources.

Key populations are central to the HST program, supported by contributions from CSOs, key population-led organizations, and key population-led services. Additionally, raising awareness of HIV infection among the general population, particularly adolescent boys and girls who are more vulnerable to infection, is a key objective of HST, achieved through various digital platform models.

## RECOMMENDATIONS

We recommend that HST should be considered an additional intervention in HIV programs to end AIDS by 2030. Its user-friendly design and potential to reduce stigma can enhance individuals’ awareness of HIV status and facilitate timely access to ART and care. For the successful introduction of HST, we recommend that the following conditions must be met.

We recommend that HST should be considered an additional intervention in HIV programs to end AIDS by 2030.

1. High sensitivity and specificity of the tests.
2. Established referral pathways to confirmation tests and ART services.
3. Affordable costs, whether covered by insurance or borne by individuals.

Legal barriers associated with facility-based testing can be overcome through the openness of national regulatory authorities and a shared goal of ending AIDS in the country.

## CONCLUSION

In the context of a well-established HIV program in Thailand, national commitments to ending AIDS, and the fact that 10% of total PLHIV in Thailand were unaware of their HIV status, we analyzed the policy of integrating HST into the benefit package, along with pilot programs and systems design. Following the inclusion of HST in the UHC, over 166,000 users received testing kits, leading to the detection of positive cases and their enrollment in the universal ART program.

## Notes

**Peer Reviewed**

**First Published Online:** December 11, 2024.
